# Mitochondrial Retrograde Signaling Contributes to Metabolic Differentiation in Yeast Colonies

**DOI:** 10.3390/ijms22115597

**Published:** 2021-05-25

**Authors:** Vítězslav Plocek, Kristýna Fadrhonc, Jana Maršíková, Libuše Váchová, Alexandra Pokorná, Otakar Hlaváček, Derek Wilkinson, Zdena Palková

**Affiliations:** 1 Department of Genetics and Microbiology, Faculty of Science, Charles University, BIOCEV, 12800 Prague, Czech Republic; v.plocek@email.cz (V.P.); krispodholova@gmail.com (K.F.); jana.rusinova@natur.cuni.cz (J.M.); derek.wilkinson@natur.cuni.cz (D.W.); 2 Institute of Microbiology of the Czech Academy of Sciences, BIOCEV, 14220 Prague, Czech Republic; vachova@biomed.cas.cz (L.V.); pokorna@biomed.cas.cz (A.P.); hlavacek@biomed.cas.cz (O.H.)

**Keywords:** mitochondrial retrograde signaling, yeast colonies, colony development and differentiation, *Saccharomyces cerevisiae*, proteomic analysis

## Abstract

During development of yeast colonies, various cell subpopulations form, which differ in their properties and specifically localize within the structure. Three branches of mitochondrial retrograde (RTG) signaling play a role in colony development and differentiation, each of them activating the production of specific markers in different cell types. Here, aiming to identify proteins and processes controlled by the RTG pathway, we analyzed proteomes of individual cell subpopulations from colonies of strains, mutated in genes of the RTG pathway. Resulting data, along with microscopic analyses revealed that the RTG pathway predominantly regulates processes in U cells, long-lived cells with unique properties, which are localized in upper colony regions. Rtg proteins therein activate processes leading to amino acid biosynthesis, including transport of metabolic intermediates between compartments, but also repress expression of mitochondrial ribosome components, thus possibly contributing to reduced mitochondrial translation in U cells. The results reveal the RTG pathway’s role in activating metabolic processes, important in U cell adaptation to altered nutritional conditions. They also point to the important role of Rtg regulators in repressing mitochondrial activity in U cells.

## 1. Introduction

**Figure 1:**
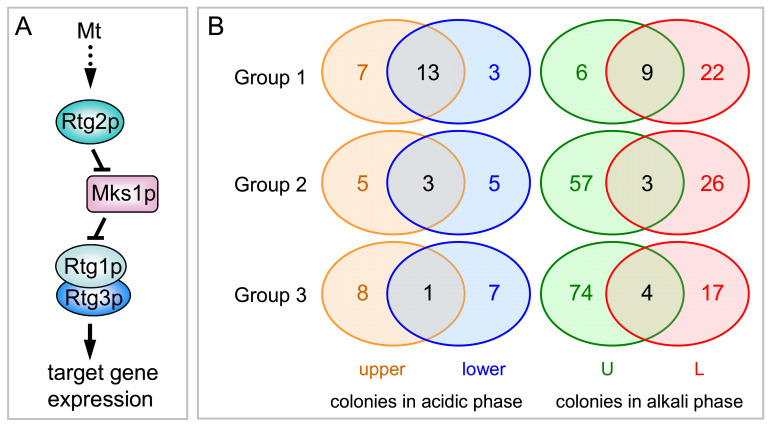
Simple scheme of standard RTG pathway regulation (**A**) and overview of genes in three groups, regulated in different ways in yeast colonies by the RTG-pathway (**B**). (**A**) The diagram shows the positions of Rtg activators and the Mks1p repressor in the standard pathway. Mt, mitochondria. (**B**) Numbers indicate genes induced by Rtg1p and repressed by Mks1p (Group 1); genes induced only by Rtg1p (Group 2) and genes repressed by Rtg1p (Group 3). Orange, upper cells; blue, lower cells (acidic-phase colonies). Green, U cells; red, L cells (alkali-phase colonies).

Mitochondria in eukaryotic cells provide key metabolic processes, energy production, cellular redox state, and a number of signaling processes affecting overall cell physiology. Disorders in various mitochondrial metabolic and signaling processes often lead to severe disorders in mammals [1]. Changes in mitochondria, whether caused by mitochondrial damage or changes in the conditions to which the cell is exposed, can activate cellular responses by a mechanism called “mitochondrial retrograde signaling”, where changes in mitochondria cause extensive changes in nuclear gene expression to help the cell adapt to the new conditions [2]. Molecular mechanisms of retrograde signaling in mammals are poorly understood, but such signaling is often associated with changes, typical of cancer disorders, including activation of enzymes involved in aerobic glycolysis [1]. The most studied system of retrograde signaling (the RTG pathway) in yeast *Saccharomyces cerevisiae* includes activators—a heterodimeric transcription factor composed of the Rtg1p and Rtg3p proteins and the Rtg2p protein, which registers mitochondrial signal(s) by an unknown mechanism [3]. In addition to RTG pathway activators, several repressors of this pathway have been identified, including the key repressor Mks1p, with which Rtg2p competes (Figure 1A) [4,5]. Negative regulation of the RTG pathway by the TORC1 protein kinase complex has also been described [3,6,7]. The *CIT2* gene encoding the peroxisomal isoform of citrate synthase hasf been identified as a typical target of the RTG pathway, and is often used as a marker of RTG pathway activation. Activation of peroxisomal functions and anaplerotic reactions, including the glyoxylate cycle, seem to be the major cellular response elicited by RTG signaling in individual liquid culture-grown cells and their mutants with mitochondrial disorders (petite strains) [8,9,10].

Unlike yeast cells in liquid media, which behave as individuals, cells within multicellular populations (colonies, biofilms) acquire various properties, typical of multicellular organisms, including the ability to differentiate and form specialized cell subpopulations that are specifically localized within the multicellular structure [11,12]. Recently, we found that RTG signaling is important for the development and differentiation of yeast colonies, and we identified three branches of RTG signaling, which all require RTG activators and the Mks1p repressor, but activate different targets [13]. Colonies grown on a complex respiratory medium pass through several developmental stages, during which they change the pH of their surroundings from acidic to alkali and vice versa. In the alkali phase, colonies produce volatile ammonia (which acts as a signaling molecule) and colony cells differentiate into two main distinct cell subpopulations—U cells in the upper and L cells in the lower colony regions [14,15,16,17]. These two cell subpopulations differ in their metabolism and regulation [14,17,18]. RTG signaling regulates expression of different marker genes in U cells and L cells, and further diversification of cellular response to this signaling has been described in upper and lower L cells. The *CIT2* gene is a typical target, regulated by the RTG pathway in upper L cells. This gene is not activated in U cells, where other RTG targets—*ATO2* and *ADY2* (*ATO1*) genes associated with ammonia signaling, were identified. Neither is it activated in lower L cells, where the RTG pathway is essential for the survival of these cells [12,13]. Typical U and L cell differentiation and RTG regulation are similar in microcolonies (colonies arising from a single cell) and in giant colonies formed from a drop of cell suspension [13,17].

In this work, we analyzed proteomes of cell subpopulations separated from colonies of strains, harboring different mutations in genes for RTG-signaling components, during two distinct stages of their development. Proteomic data, supplemented by microscopic analyses along with detailed quantification of selected proteins and mRNA, allowed us to gain a comprehensive view of the processes, regulated by the RTG pathway in cell subpopulations of colonies at various stages of their development.

## 2. Results

### 2.1. Genome-Wide Proteomic Analysis of Colony Cell Subpopulations

Giant colonies of strain BY4742 (wt) and knockout (KO) strains BY-*rtg1*, BY-*mks1*, BY-*rtg1mks1* and BY-*rtg2mks1* (Table 1) were cultivated on GMA plates until they reached the acidic (5-day-old) or alkali (15-day-old) developmental phase. Then, two cell subpopulations were harvested, upper and lower cells from the acidic-phase colonies and U and L cells from alkali-phase colonies. Lysates were prepared from each cell sample and proteomes were analyzed using a label-free LC-MS/MS method. 

**Figure 2:**
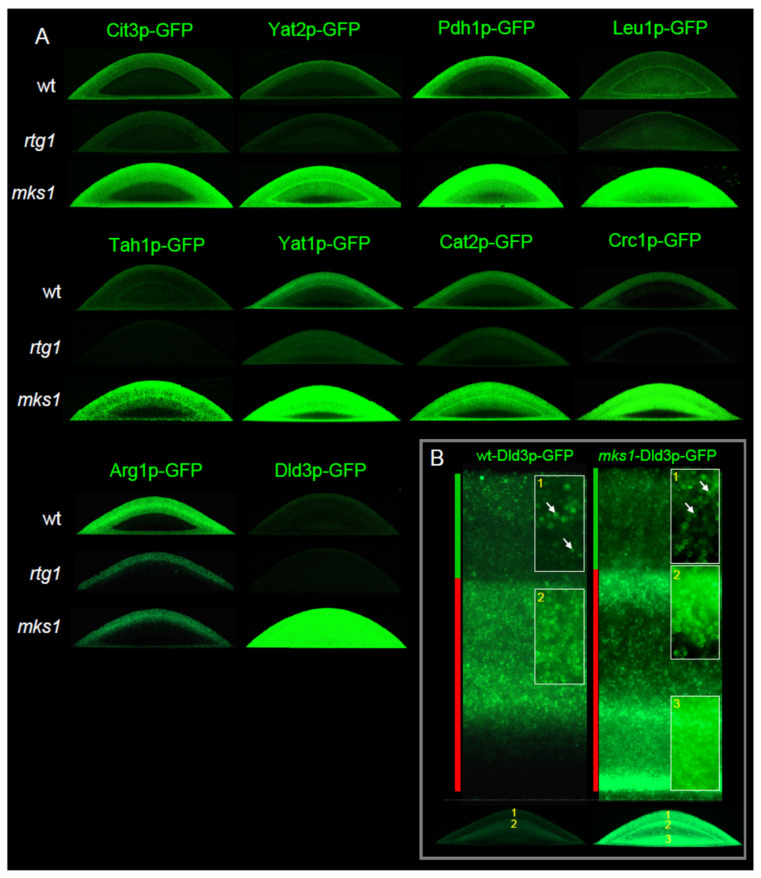
Levels of selected Group 1 proteins fused with GFP within alkali-phase differentiated colonies of wt and KO strains. (**A**) Localization of the indicated protein is visualized as green fluorescence on vertical colony cross sections of 4-day-old microcolonies in the alkali phase. The same microscopy setup was used for all colonies of wt, *rtg1* and *mks1* strains producing the indicated fusion proteins. Thus, fluorescence intensity roughly reflects differences in the level of the particular protein in colonies of the three strains. (**B**) Cross-sections of central regions of BY-Dld3p-GFP and BY-*mks1*-Dld3p-GFP microcolonies in later alkali phase showing cellular localization of Dld3p-GFP in higher magnification. Arrows indicate localization of free GFP (a degradation product of Dld3p-GFP) in vacuoles. Different exposure times were employed during microscopy of the two strains to show details of Dld3p-GFP localization in spite of strong differences in Dld3p-GFP levels in their colonies as shown in (**A**).

**Figure 3:**
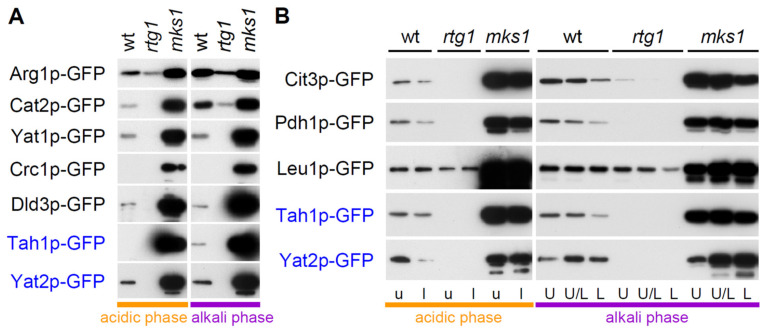
Comparison of amounts of selected Group 1 proteins in colonies of wt and KO strains. (**A**) Levels of selected Group 1—GFP proteins in 2-day-old acidic- and 4-day-old alkali-phase microcolonies formed by wt and KO strains. (**B**) Levels of selected Group 1—GFP proteins in upper (u) and lower (l) cells from 5 day-old acidic-phase giant colonies and in U, U/L (cells close to the border between U and L cells) and L cells from 15-day-old giant colonies formed by wt and KO strains were determined by Western blot. In blue, protein levels were analyzed in both microcolonies and giant colonies. Loading controls are in Figures S1 and S2.

**Figure 4:**
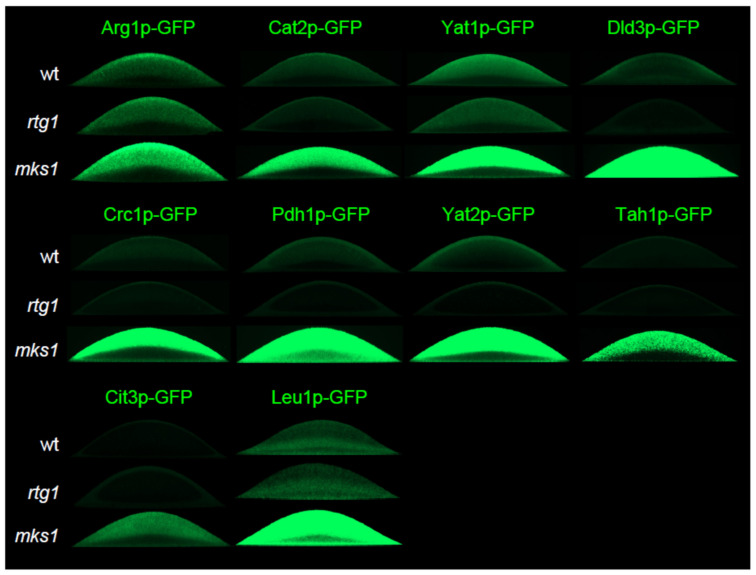
Levels of selected Group 1 proteins fused with GFP within acidic-phase colonies of wt and KO strains. Localization of the indicated protein is visualized as green fluorescence on vertical colony cross sections of 2-day-old microcolonies. The same microscopy setup was used for all colonies (wt, *rtg1* and *mks1*) producing the indicated protein. Thus, fluorescence intensity roughly reflects differences in the level of the particular protein in colonies of the three strains.

In the RTG pathway (Figure 1A), Rtg2p functions upstream of Mks1p, and thus the combined *rtg2mks1* deletion should affect RTG signaling similarly to the *mks1* deletion itself. In contrast, Rtg1p functions downstream of Mks1p in the pathway, and thus the combined *rtg1mks1* deletion should affect RTG signaling similarly to the *rtg1* deletion itself. Therefore, we analyzed proteomics datasets to identify proteins, significantly enriched or reduced in both the wt versus BY-*rtg1* and wt versus BY-*rtg1mks1* subpopulations, as well as those, enriched or reduced in the wt versus BY-*mks1* and wt versus BY-*rtg2mks1* subpopulations. The identification of enriched/reduced proteins in such a pairwise analysis of strains with similar behavior strongly reduces non-specific background (especially in the case of less abundant proteins) and eliminates differences, unrelated to RTG signaling. In addition, we compared the abundance of proteins in the upper/U and lower/L cells of wt colonies to select groups of proteins, more abundant in upper/U or lower/L cells, respectively. Further grouping of proteins selected in the pairwise analysis led to identification of three main protein groups with different patterns of regulation by Rtg regulators (Figure 1B). To validate proteomic data, we constructed wt strains producing GFP-tagged versions of selected identified proteins and subsequently KO strains with either the *RTG1* or *MKS1* gene deleted. The level of GFP-tagged proteins in colonies was monitored in situ by fluorescence microscopy and in cells separated from colonies by Western blot (Figure 2, Figure 3 and Figure 4). 

### 2.2. Group 1 Proteins Are Regulated by the RTG Pathway in a Standard Way

Group 1 includes proteins, whose abundance was significantly higher in cell subpopulations of wt versus both BY-*rtg1* and BY-*rtg1mks1* and, concurrently, lower in wt versus BY-*mks1* and BY-*rtg2mks1*. Expression of these proteins should thus be conventionally regulated by the RTG pathway, with Rtg1p as a positive regulator and Mks1p as a repressor. In alkali-phase colonies, 15 proteins belonging to this group were identified in U cell-samples and 31 in L cell-samples (Figure 1B, Table S1A). From these, nine proteins (Crc1p, Oac1p, Ato2p, Yat1p, Cit3p, Yat2p, Pdh1p, Leu4p and Leu1p) were identified in both the U and L cell datasets, with Ato2p and Cit3p significantly more abundant in U cells, compared with L cells of wt colonies (columns U and V). The other seven proteins were similarly expressed by both U and L cells of wt colonies. Of six proteins, specifically identified in the U cell dataset (Adh4p, Tah1p, Err3p, Fdh1p/Fdh2p, Cat2p and Icl2p), five were more abundant in U than L cells of wt colonies. The level of all 6 proteins was markedly enhanced in L cells by *MKS1* gene deletion. Similarly, deletion of *MKS1* increased the levels of nine proteins that are regulated by the RTG pathway in both U and L cells, more-so in L cells than in U cells. Of 22 proteins of Group 1 with typical RTG regulation only in L cells, 3 proteins (Cit2p, Dld3p, Ree1p) exhibit significantly higher expression in L than U cells of wt colonies. Of these proteins only Dld3p was strongly repressed by Mks1p in U cells; Mks1p repression of the others was mild. The other 14 proteins exhibited higher expression in U than L cells and 5 were equally expressed. None of these 19 proteins was repressed in U cells by Mks1p, but Sry1p accumulation was strongly enhanced by the presence of Mks1p and levels of the other 5 proteins were only moderately enhanced. 

Strains with selected Group 1—proteins identified in alkali-phase colonies (Cit3p, Yat2p, Pdh1p, Leu1p, Tah1p, Yat1p, Cat2p, Crc1p, Arg1p and Dld3p) labeled with GFP, as well as their *rtg1*Δ and *mks1*Δ derivatives, were further analyzed. Fluorescence and confocal microscopy of cross-sections of 4-day-old alkali-phase microcolonies (Figure 2) of these strains together with Western blots (Figure 3) confirmed typical RTG-pathway regulation of these proteins and provided additional information on their level in colony cell subpopulations. In addition to Cit3p-GFP and Cat2p-GFP identified in proteomics to be more abundant in U than in L cells of 15-day-old giant colonies, Yat2p-GFP, Pdh1p-GFP, Yat1p-GFP, Crc1p-GFP and Arg1p-GFP exhibited similar expression patterns in alkali-phase microcolonies (Figure 2A). Leu1p-GFP levels in U and L cells were similar. Tah1p and Dld3p levels in wt microcolonies were low and fluorescence levels were similar to background autofluorescence, but both proteins were detected in wt colonies by Western blot (Figure 3A). Dld3p exhibited a similar concentration profile in the proteomics analysis as Cit2p, which is present in upper cells of acidic-phase colonies but disappears from U cells after U/L cell differentiation while continuing to be present in upper L cells [13]. A detailed microscopic analysis of Dld3p-GFP cellular localization in microcolonies of the wt and *mks1*Δ strains in the later alkali phase (~5-day-old), revealed a pattern, similar to that of Cit2p-GFP in U cells and upper L cells (Figure 2B). Cit2p-GFP abundance does not increase in lower L cells of the *mks1*Δ strain. In contrast, the Dld3p-GFP level also strongly increased in lower L cells upon *MKS1* deletion. Western blots of Cit3p-GFP, Pdh1p-GFP, Leu1p-GFP, Tah1p-GFP and Yat2p-GFP cell subpopulations separated from 15-day-old alkali-phase giant colonies (Figure 3B) showed higher level of Cit3p-GFP, Pdh1p-GFP and Tah1p-GFP in U cells compared to L cells of wt colonies. Leu1p-GFP levels were similar in both cell types and Yat2p-GFP levels were higher in L than in U cells. These comparisons showed that all targets tested are regulated by the RTG pathway in both giant colonies and microcolonies with a largely similar pattern of expression in differentiated cells. An exception is Yat2p with a higher level in U cells of microcolonies but a slightly higher level in L cells in giant colonies in alkali phase. 

In acidic-phase colonies (Figure 1B, Table S1B), overlap between Group 1 proteins, identified in proteomics of upper and lower cell-samples, was higher than between U and L cells of differentiated alkali-phase colonies (Table S1A). In acidic-phase upper cells 20 proteins, and in lower cells 16 proteins, belong to this group. Thirteen of these proteins (Pdh1p, Crc1p, Gdh3p, Ady2p, Cit3p, Cit2p, Icl2p, Yat2p, Yat1p, His4p, Mae1p, Acs1p and Cat2p) were identified in both cell types and regulated with similar efficiency by both Rtg1p and Mks1p. Accordingly, only two (Cit3p, His4p) of these proteins exhibited a slightly higher level in upper than in lower cells while others were equally abundant in each. Six of seven Group 1 proteins of that were identified only in upper cells (Arg1p, Leu4p, Dld3p, Hom3p, Sps100p, Lys4p, Aco2p) and two of the three identified only in lower-cells (Ald5p, Ade17p and Nde2p), were moderately repressed in upper cells by Mks1p. Dld3p was strongly Mks1p-repressed in both upper and lower cells. Microscopy and Western blotting analysis of acidic-phase microcolonies of strains with selected targets labeled with GFP (Figure 3A and Figure 4) confirmed RTG pathway regulation and showed relatively moderate (or even undetectable) amount of these proteins in microcolonies together with their strong induction in upper colony regions due to *MKS1* deletion. 

Altogether, we identified 40 proteins belonging to Group 1, whose levels were conventionally regulated by the RTG pathway in either both acidic and alkali colonies (22 proteins) or only in acidic (3 proteins) or only in alkali (15 proteins) colonies.

### 2.3. Group 1 Proteins Are Regulated by the RTG Pathway at the Level of mRNA Expression

**Figure 5:**
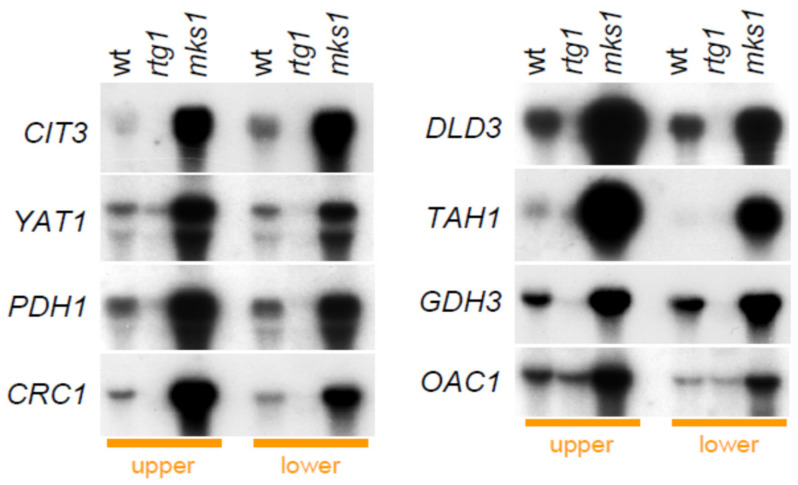
Comparison of levels of mRNAs encoding selected *Group 1* proteins in colonies of wt and KO strains. Amounts of mRNAs encoding particular proteins in cells of upper and lower fractions of 5-day-old giant colonies formed by wt, BY-*rtg1* and BY-*mks1* strains, detected by Northern blot. Loading controls are in Figure S3.

Next, we investigated whether Group 1 proteins are regulated by the RTG pathway at the transcriptional level (mRNA level). We separated cells from upper and lower parts of 5-day-old acidic-phase giant colonies of wt, BY-*rtg1* and BY-*mks1* strains and analyzed mRNA of selected genes (*CIT3*, *YAT1*, *PDH1*, *CRC1*, *DLD3*, *TAH1*, *GDH3*, *OAC1*) by Northern blot (Figure 5). In all cases, the level of mRNA was lower in the BY-*rtg1* and higher in the BY-*mks1* strain as compared to wt, thus confirming mRNA expression regulation via the RTG pathway. Levels of most of the mRNAs were not significantly different between upper and lower cells in colonies of the wt strains, whereas *MKS1* deletion often enhanced mRNA levels more in upper than in lower cells. 

### 2.4. Two Other Groups of Proteins Are Regulated by Rtg Proteins Independently of Mks1p Repression

Group 2 includes proteins whose levels were decreased in both BY-*rtg1* and BY-*rtg1mks1* cells as compared to wt, but were not increased by *MKS1* deletion in BY-*mks1* and BY-*rtg2mks1* cells. A total of 60 and 29 proteins belonging to this group were identified in U and L cells, respectively, in alkali-phase colonies (Figure 1B, Table S2A). Of these, only three proteins were identified in both the U and L cell datasets, whereas others were specific either to U or L cells. The expression of 20 out of 60 and 6 out of 29 of these proteins also decreased after the deletion of *MKS1* (in both BY-*mks1* and BY-*rtg2mks1* cells). U cell specific proteins belonging to Group 2 were enriched (*p* < 0.01) for Gene Ontology (GO) terms related to amino acid metabolic (mostly biosynthetic) processes, mainly enzymes involved in biosynthesis of arginine, glutamine, aspartate, lysine and sulfur amino acids (Table S2A). Most (48) of these proteins were more abundant in U than in L cells of wt colonies. The abundance of some of these proteins was not affected in BY-*mks1* and BY-*rtg2mks1* cells, some others proteins abundances were lower in these strains. L cell proteins of Group 2 were enriched (*p* < 0.01) only for GO terms related to vitamin (mainly thiamine) biosynthetic processes. The levels of all identified vitamin-related proteins also decreased upon deletion of *MKS1*. Only 13 proteins of acidic-phase colonies belong to Group 2 (Figure 1B). Eight were identified in upper and eight in lower cells, including three proteins, detected in both upper and lower cells. No significant GO term category of has been identified and most of these Group 2 proteins were also identified in alkali-phase colonies (Table S2B).

Group 3 includes proteins whose levels were increased in both BY-*rtg1* and BY-*rtg1mks1* cells as compared to the wt (Figure 1B, Table S3). A small number of these proteins also increased in concentration due to *MKS1* deletion. 78 proteins were identified in U cells of alkali-phase colonies (Table S3A), 9 of which increased in concentration due to *MKS1* deletion. Twenty-one proteins were identified in L cells, including two proteins, the level of which decreased due to *MKS1* deletion. Only four proteins of Group 3 were identified in both U and L cells. Sixty-eight proteins with increased abundance in U cells upon *RTG1* deletion, but not upon *MKS1* deletion, were strongly enriched for the GO Process and Component terms “mitochondrial translation”, “mitochondrial gene expression” and “mitochondrial ribosome” (Table S3A). All proteins in these GO categories were only repressed by Rtg1p in U cells and did not exhibit any other Rtg1p and/or Mks1p regulation in either U or L cells (Table S3A). An additional GO category “long-chain fatty acid biosynthetic process” also fulfilled significance criteria (*p* < 0.01) for only Rtg1p-regulated proteins in U cells. No significant GO term category was identified in Group 3 proteins in L cells. 

In acidic-phase colonies only 16 proteins belong to Group 3 (Figure 1B, Table S3B); nine in upper and eight in lower cells with only one protein identified in both. Only two proteins were also repressed by Mks1p. Group 3 proteins in acidic-phase colonies were enriched (*p* < 0.01) only for the GO Component term “mitochondrion”.

## 3. Discussion

Previous findings indicate the importance of mitochondria and the RTG pathway in the development and differentiation of yeast colonies [12,13,14,17]. In particular, Rtg-/Mks1p—dependent regulation of expression of specific proteins was identified in specific subpopulations—Ato2p and Ady2p expression in U cells and Cit2p expression in upper L cells [13]. These two RTG branches (Ato- and Cit-) are thus differently regulated in relation to TORC1, one of the described repressors of the RTG pathway. The Ato- branch in U cells is active in the presence of active TORC1, while the Cit-branch in L cells is active under conditions of inactive TORC1 [13]. The situation in L cells is thus consistent with described inhibition of the RTG pathway by TORC1 [3]. In acidic phase colonies (prior to U/L cell differentiation) TORC1 is inactive in all cells and only Cit2p is induced via the RTG pathway in upper parts of these colonies [13]. In this work, we aimed to identify other important proteins, the expression of which is controlled by the RTG pathway in specific cell subpopulations, and thus to identify cellular processes that the RTG pathway controls in colonies. The basis was an extensive analysis of proteomes of differentiated colony cells in two developmental phases (acidic and alkali) followed by subsequent microscopic analyses of selected GFP-labeled proteins in colonies and their more detailed quantification. To increase the significance of conclusions, arising from the proteomic data, we took advantage of the fact that two pairs of strains with different combinations of RTG-pathway gene deletions could be expected to exhibit similar phenotypes, in terms of pathway activity, as compared with the wt strain. Therefore, in subsequent analyses and interpretation of results, we took into account only those proteins that were significantly regulated in both members of the relevant pair of mutants versus the wt. In this way, we revealed three main groups of proteins, expression of which was regulated differently in colonial cells. The first group included proteins with conventional RTG pathway regulation—induction by Rtg1p and repression by Mks1p. The second group included proteins that were induced by Rtg1p but were not significantly repressed by Mks1p in the same cells. The third group included proteins that were repressed by Rtg1p. 

Group 1, classically RTG-regulated proteins were enriched for GO terms, mainly related to various metabolic processes. Proteomic analysis, along with detailed microscopic and Western blot analyses of selected GFP-labeled proteins, showed that most of these proteins localize predominantly to U cells in differentiated alkali-phase colonies, and thus had similar expression profiles to the previously identified Ato-branch of RTG signaling [13] and are activated by RTG in the presence of active TORC1. In contrast, only Dld3p and Ree1p had similar proteomic profiles to the previously identified Cit2p, being more abundant in L than in U cells of wt colonies. Like Cit2p, Dld3p was expressed in the upper parts of acidic-phase colonies and then degraded in U cells but remained in L cells of alkali-phase colonies, consistent with inactive TORC1. In contrast to Cit2p, Dld3p was also present in lower L cells. Several Group 1 proteins were also detected in younger acidic-phase colonies, where proteomics showed no significant differences between upper and lower cell abundance. However, analyses of the amounts of selected proteins (Western blot, microscopy) mostly showed slightly higher amounts of these proteins in upper cells in both microcolonies and giant colonies in acidic phase. Deletion of *MKS1* usually strongly increased levels of these proteins especially in upper cells of acidic-phase colonies (similar to Ato proteins), suggesting strong Mks1p repression in conditions of inactive TORC1, whereas their derepression in U cells in the absence of Mks1p was less pronounced than in L cells. The level of Dld3p-GFP was also strongly increased in lower cells. Analysis of mRNA of selected genes confirmed that Rtg/Mks1p-dependent effects, detected at protein level, correlate with respective mRNA-level effects. These results confirmed that RTG-pathway regulation of identified proteins occurs mostly at the transcriptional level. 

The proteins included in Group 2 were also positively controlled by Rtg1p, but unlike Group 1, were not significantly repressed by Mks1p in the same cells. In contrast, in some cases the absence of Mks1p led to a reduction in protein levels, as did the absence of Rtg1p. The largest cluster of such proteins was identified in U cells, where they are mainly involved in the biosynthesis of some amino acids. Although these proteins are not repressed by Mks1p in U cells, deletion of *MKS1* slightly increased the amount of many of them in L cells. It is known from previous findings that the amount of Cit2p in U cells, unlike L cells, is not increased by deletion of *MKS1*, although Cit2p is classically regulated by the RTG pathway [13]. It is therefore possible that Group 2 U cell-upregulated proteins, related to amino acid metabolism, are conventionally regulated by Rtg/Mks1p, but that Mks1p repression does not operate (or is less effective) in U cells and so expression of these proteins is Mks1p-repressed in L cells but not U cells of wt colonies. The fact that many of these proteins are significantly more highly expressed in U than in L cells of wt colonies supports this possibility. The number of Group 2 proteins, identified in L cells was half that, identified in U cells, and these proteins were not expressed upon *MKS1* deletion in any cell type. The only significant functional GO group identified among these proteins related to the biosynthesis of vitamins, especially thiamine. Only 13 proteins in acidic-phase colonies belong to Group 2, and although no significant functional group was identified, the genes for thiamine biosynthesis clearly belonged to this group, even in the acidic phase.

**Figure 6:**
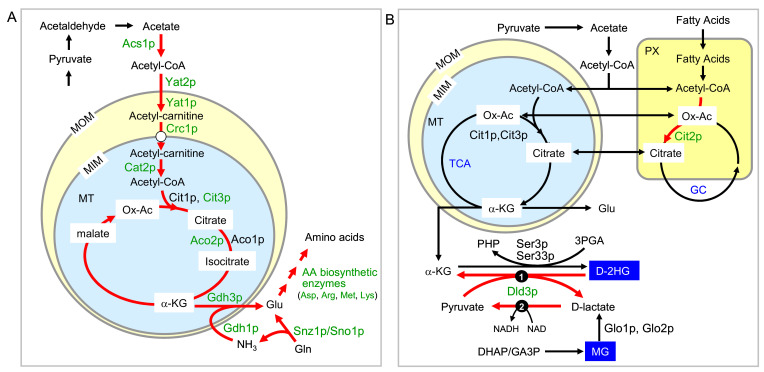
Scheme of metabolic functions, induced by RTG pathway in U cells (**A**) and L cells (**B**). Proteins, the level of which increased upon Rtg protein activation, are in green, and related pathways in red. MOM, mitochondrial outer membrane; MIM, mitochondrial inner membrane; MT, mitochondria; PX, peroxisome; TCA, TCA cycle; GC, glyoxylate cycle; Ox-Ac, oxaloacetate; α-KG, α-ketoglutarate; PHP, 3-phosphohydroxypyruvate; 3PGA, 3-phosphoglycerate; D-2HG, D-2-hydroxyglutarate; MG, methylglyoxal; DHAP, dihydroxyacetone phosphate and GA3P, glyceraldehyde 3-phosphate. Reaction 1—predicted function of Dld3p in D-2HG metabolism; reaction 2—predicted function of Dld3p in MG metabolism.

Which processes may be activated by Rtg proteins (whether or not repressed by Mks1p) in differentiated colony subpopulations? In U cells, a prominent subset of Rtg-activated proteins consists of proteins enabling the synthesis of acetyl-CoA, its transport to mitochondria and its consumption via the TCA cycle (Figure 6). One route by which acetyl-CoA (produced from acetate or by β-oxidation of fatty acids) reaches the mitochondria involves the carnitine shuttle, followed by acetyl-CoA consumption through the TCA cycle. The second requires extra-mitochondrial Cit2p to produce citrate from oxaloacetate and acetyl-CoA. Citrate then either enters the mitochondria to be fed into the TCA cycle or is used in the glyoxylate cycle [3,19,20,21,22]. The first acetyl-CoA route appears to run in U cells under the control of the RTG pathway (Figure 6A), as the Acs1p, Cat2p, Yat1p, Yat2p, Cit3p and Aco2p proteins are characteristic of these cells and their expression depends on Rtg proteins. Expression of genes for some of these proteins has previously been shown to be elevated in respiratory-deficient cells, but without being linked to RTG pathway regulation [3]. Another subset of Rtg-regulated proteins in U cells is involved in nitrogen (NH_3_) recycling and glutamate formation, such as Gdh3p and Gdh1p forming glutamate from α-ketoglutarate and NH_3_ and Sno1p/Snz1p putative glutaminases converting glutamine to glutamate and NH_3_ (Figure 6A). These data, together with identification of a large group of proteins involved in amino acid biosynthesis (among Group 2 proteins), suggest that regulating the use of nutritive compounds (waste products and/or compounds provided by L cells [14,18]) for synthesis of amino acids could be a major role of the RTG pathway in U cells (Figure 6A). In addition, higher amounts of both Cit3p and Icl2p suggest that U cells may cope better with some side-products of amino acid metabolism, such as propionyl-CoA (which may arise from degradation of branched chain amino acids), by its removal via the methyl citrate pathway and pyruvate formation [9,23,24]. 

In contrast to U cells, L cells express Cit2p and probably use the second acetyl-CoA route preferentially (Figure 6B). Cit2p could also contribute to consumption of propionyl-CoA (if present) in peroxisomes, but this would lead to the accumulation of toxic 2-methylcitrate [23,24]. Besides Cit2p, the RTG pathway controls Dld3p expression in L cells. Both genes, *DLD3*, encoding a cytoplasmic D-lactate dehydrogenase, and *CIT2* for citrate synthase, are genes classically controlled by the RTG pathway under different conditions and different cultures [25,26]. Dld3p could be involved in detoxification of D-2-hydroxyglutarate (D-2HG) [27] and methylglyoxal [28]. Major intracellular sources of D-2HG are Ser3p and Ser33p 3-phosphoglycerate dehydrogenases that catalyze the first step in serine and glycine biosynthesis and have additional α-ketoglutarate reductase activity, oxidizing 3-phosphoglycerate and reducing α-ketoglutarate to D-2HG. Dld3p was recently shown to have transhydrogenase activity, oxidizing D-2HG to α-ketoglutarate and reducing pyruvate to D-lactate [27]. MG arises non-enzymatically from dihydroxyacetone phosphate/glyceraldehyde 3-phosphate [28,29,30] and accumulates under conditions of impaired glucose metabolism or oxidative stress. In yeast, the cellular MG level peaks during the diauxic shift. MG is converted to D-lactate by Glo1p and Glo2p or Glo4p. Cytosolic Dld3p oxidizes D-lactate to pyruvate, which can participate in gluconeogenesis [28]. These findings are consistent with L cell characteristics as stressed cells with active gluconeogenesis [14,18]. Thus, according to the literature, yeast Dld3p can catalyze the bidirectional conversion of D-lactate to pyruvate and back in the cytosol. However, a recently postulated model in mammals hypothesizes that only the first part of the lactate-pyruvate cycle—the reduction of pyruvate to L-lactate (the end product of mammalian glycolysis)-takes place in the cytosol, while the reverse reaction—oxidation of L-lactate to pyruvate-takes place in the mitochondrial matrix [31]. It is therefore possible that in yeast, oxidation of D-lactate to pyruvate also localizes to the mitochondria, where a minor form of lactate dehydrogenase Dld2p is present. However, the level of Dld2p is not controlled by the RTG pathway (not shown). 

The third group consists of proteins, negatively regulated by Rtg1p, most of which are not affected by the presence or absence of Mks1p. Like Group 2 proteins, Group 3 proteins are almost absent from acidic-phase colonies, and were most abundant in U cells. Of the 78 proteins of this group, identified in U cells, 55 proteins are only repressed by Rtg1p specifically in U cells (they show no other Rtg/Mks1p dependent regulation in any other cells). In colonies of the wt strain, most of these proteins have elevated levels in L cells as compared to U cells. GO functional analysis revealed with high significance that 31 of these proteins are related to mitochondrial functions, including proteins involved in mitochondrial translation and ribosome composition and function.

The results of this work confirmed the importance of mitochondrial regulation in colonies and identified processes, specifically regulated by Rtg factors. In comparison with the previously identified Ato- and Cit2- specific pathways, we showed that most proteins classically controlled by the RTG pathway have a similar expression and regulation profile to proteins of the Ato-branch of the pathway and that most Rtg1p-dependent regulations occur in U cells under conditions of active TORC1 and high intracellular glutamine [14]. A relatively lower number of genes is regulated by this pathway in L cells and such regulations are often already apparent in younger colonies in acidic phase. In addition to genes, classically regulated via Rtg induction and Mks1p repression, a significant number of genes, mainly genes involved in amino acid biosynthesis, are regulated in U cells only by Rtg1p. Mks1p moderately regulates some of these genes only in L cells. The main effect of TORC1 (and glutamine) on RTG signaling (often measured via expression of *CIT2*, the most commonly used marker of the RTG pathway) presumably occurs via phosphorylation of the Mks1p repressor, which in complex with Bmh1p/Bmh2p inhibits the Rtg1p-Rtg3p heterodimeric transcriptional activator. In low glutamine and inactive TORC1 conditions, Mks1p phosphorylation is reduced or eliminated leading to RTG activation involving Rtg2p. The model assumes that different levels of Mks1p phosphorylation, due to glutamine/TORC1 activity, contribute to the intensity of the RTG response under different nutrient conditions (e.g., different nitrogen sources) [6]. Phosphorylation of Rtg3p may or may not reflect the level of Mks1p phosphorylation, TORC1 activity, and activation of Cit2p target protein expression. In most cases, activation of the RTG response (measured via Cit2p expression) is associated with dephosphorylation of Rtg3p, but its hyperphosphorylation after prolonged inhibition of TORC1 by rapamycin has also been described. However, such hyperphosphorylation did not reduce Cit2p expression [32]. RTG regulation (including Mks1p repression) may also be TORC1 independent [33], and involvement of Rtg2p (a key activator of the pathway) assumes a signal from dysfunctional (or less functional) mitochondria [34,35]. These and other findings show that the RTG pathway responds in different ways to external conditions, and that many of the mechanisms involved in this response have not yet been elucidated. In colonies, Cit2p expression roughly correlates with inactive TORC1 (L cells and upper cells of acidic colonies), but the vast majority of RTG-dependent activations occur under conditions of active TORC1 (and high glutamine) in U cells either repressed or not repressed via Mks1p. These findings imply that, under certain metabolic conditions, part of the RTG response occurs independently of TORC1 and is, presumably, only partially dependent on Mks1p, implying another hitherto unknown RTG regulation by a specific cell state. Because U cells exhibit a number of non-standard regulations, including parallel TORC1 activity, active autophagy, active Gcn4p, and others [14,36], atypical regulation of the RTG pathway in relation to TORC1/gln is another example of the metabolic uniqueness of these cells. One possible explanation is that Rtg2p (which is a key activator in both the Cit- and Ato- branches of colonial RTG signaling, [13] activates the RTG pathway partially independently of Mks1p and thus part of the pathway is constitutively active in U cells. However, since some typical RTG responsive genes (*CIT2*, *DLD3*) are excluded from this activation, further (as yet unidentified) regulations are likely, which contribute to the specificity of RTG pathway-induced targets in different colony cell subpopulations.

Positive Rtg factor regulation in U cells is related to metabolic (mostly biosynthetic) processes together with processes involved in transport of metabolic intermediates to mitochondria (such as the carnitine shuttle), which together contribute to recycling available nutritive compounds to support the biosynthesis of amino acids, which is a typical characteristic of U cells described previously [14]. The expression of a number of genes for these proteins is elevated in respiratory-deficient cells [3], consistent with findings of decreased respiration in U cells [12,14,17,37]. Some of the Rtg-induced processes (e.g., carboxylic acid metabolism, carnitine shuttle) are already Rtg-dependent in younger colonies in the acidic phase, where they are often strongly repressed by Mks1p. In differentiated alkali-phase colonies, these processes are strongly activated by Rtg1p in U cells, where the repressive function of Mks1p is attenuated. In contrast, Mks1p repression of these processes remains strong in L cells, where the induction effect of Rtg1p is weak. In accordance, proteins belonging to these groups often exhibit higher expression in U than in L cells of wt colonies. 

In addition to the above-described, positive regulation of metabolic processes by the RTG signaling pathway, we identified a significant functional group of proteins, uniquely repressed by Rtg1p in U cells, without any Mks1p effect. Proteins of this group participate in mitochondrial translation, mainly being components of mitochondrial ribosomes. In wt colonies, most of these proteins exhibit higher accumulation in L cells than U cells. It was shown by transcriptomic studies, that expression of nuclear genes encoding mitochondrial ribosome proteins is co-regulated in yeast [38] being repressed by glucose and anaerobiosis [39,40]. The RTG pathway thus also appears to directly or indirectly contribute to the reduction of mitochondrial respiration in U cells, a prominent metabolic characteristic that these cells share with cells of solid tumors [14]. Changes in the expression of genes encoding mitoribosomal proteins have been associated with tumorigenesis in mammals [41].

## 4. Materials and Methods

### 4.1. Yeast Strains and Cultivation

All *S. cerevisiae* strains in this study (Table 1) were derived from the laboratory strain BY4742 from the Euroscarf collection. C-terminal GFP fusion strains were prepared using a GFP integrative cassette amplified by PCR from plasmid pKT127 (kanr) [42]. Gene knockouts were prepared using deletion integrative cassette amplified by PCR from plasmid pUG6 + 25 (natr) [13,43]. The primer sequences are listed in Table S4. Yeast cell transformation was performed according to [44]; 100 μg/mL nourseothricin and 400 μg/mL G418 were used to select yeast transformants. 

Giant colonies were inoculated onto the agar surface (six per plate), using 10 µL drops of 12 mg/mL cell suspension. Microcolonies were grown at an approximate density of 5 × 10^3^ per plate. All colonies were grown on respiratory GMA medium (1% yeast extract, 3% glycerol, 1% ethanol, 2% agar, and 10 mM CaCl_2_, with or without the pH indicator bromocresol purple) at 28 °C.

### 4.2. Colony Imaging Microscopy

To analyze internal structure of colonies we used two photon excitation confocal microscopy (2PE-CM) or fluorescence microscopy of thin colony cross sections as described earlier [14,45,46]. Briefly, 2PE-CM colonies embedded in agarose were cut vertically in the middle. The cut surface was placed on a coverslip and side views of internal colony structure were obtained by a confocal scanning microscope (AxioObserver.Z1) with a confocal module LSM 880 NLO and MP (Carl Zeiss, Oberkochen, Germany) with Ti:Sapphire femtosecond laser Chameleon Ultra II (Coherent, Santa Clara, CA, USA) and 25×/0.8 or 63×/1.2 water immersion LD LCI Plan- or C-Apochromat objectives, respectively. An excitation wavelength of 920 nm and emission bandwidth of 480–595 nm was used for GFP. To obtain images of whole colonies we combined two images from neighboring fields of view. For fluorescence microscopy, thin sections were prepared from colonies embedded in 3% agarose using a Leica VT1200S vibrating microtome (Leica, Wetzlar, Germany) [14,47] and observed by an Axio Observer.Z1 fluorescence microscope (Carl Zeiss, Oberkochen, Germany) equipped with an Axiocam 506 and an Apochromat 10×/0.45W objective or an Apochromat 63×/1.20 W using ZEN 2012 (blue edition) software. Filter sets for GFP fluorescence: 450–490 nm and 500–550 nm for excitation and emission, respectively. 

### 4.3. Cell Biomass Harvesting

By micromanipulation, two types of cells, upper/U and lower/L cells, were harvested from upper and lower parts of giant colonies, in the acidic and alkali developmental phases. Purity of the fractions was checked by microscopy using DIC contrast. Alternatively, the biomass of whole microcolonies was harvested. The harvested biomass was immediately frozen in liquid nitrogen and stored at −80 °C and used for proteomic, Western- or Northern-blot analyses. 

### 4.4. Proteomics Analysis

Comparison of proteomes was performed by nano LC-MS/MS analysis. Cells harvested from giant colonies were disrupted in 100 mM triethylammonium bicarbonate (TEAB), 10 mM Tris(2-carboxyethyl)phosphine, 50 mM chloroacetamide buffer containing 2% sodium deoxycholate using glass beads five-times for 20 s using a FastPrep (Thermo Fisher Scientific, Waltham, MA, USA); after the first two runs, samples were heated for 5 min at 95 °C. Protein aliquots (30 µg per sample; determined by bicinchoninic acid assay, Sigma-Aldrich, St. Louis, MO, USA) were used for MS sample preparation. Samples were further processed using SP3 beads according to [48]. Briefly, 5 µL of SP3 beads were added to 30 µg of protein in lysis buffer and made up to 50 µL with 100 mM TEAB. Protein binding was induced by addition of ethanol to 60% (*v*/*v*) final concentration. Samples were mixed and incubated for 5 min at laboratory temperature. After binding, the tubes were placed into a magnetic rack and the unbound supernatant was discarded. Beads were subsequently washed twice with 180 µL of 80% ethanol. After washing, samples were digested with trypsin (trypsin/protein ratio 1/30), acidified with TFA to 1% final concentration, and peptides were desalted using C18 disks (CDS Analytical; Oxford, PA, USA). Peptides (2 µg) from each sample were separated on Nano reversed phase columns (EASY-Spray column a 50 cm × 75 μm ID, PepMap C18, 2 μm particles using a 1 h elution gradient and analyzed in DDA mode on an Orbitrap Fusion Tribrid (Thermo Scientific, Waltham, MA, USA) mass spectrometer. For each strain and condition, three biological replicates were analyzed. Resulting raw files were processed in MaxQuant (v. 1.6.10.43) and searches performed against the latest version of *S. cerevisiae* Uniprot database and common contaminant database. Perseus (v.1.6.1.1.) and Excel 2013 were used for further analysis. Significance of protein abundance differences between two strains was determined using the unpaired two-tailed t test. *p* values of 0.05 or less were considered statistically significant. Functional categories enriched in particular comparisons of proteomes were identified using the GO term finder at SGD (https://www.yeastgenome.org/goTermFinder, accessed on 16 October 2020) (*p*-value 0.01). The proteomics data were deposited at the ProteomeXchange Consortium via the PRIDE (identifier PXD026077).

### 4.5. Determination of the Amount of GFP-Labeled Proteins by Western Blot

Western blot detection of GFP labeled proteins was performed as described [49]. Briefly, biomass harvested from microcolonies or giant colonies was disrupted using glass beads and proteins in cell lysates (4–25 µg in a well) were subjected to SDS-PAGE and proteins transferred to a PVDF membrane. GFP was detected by mouse monoclonal horseradish peroxidase (HRP)-conjugated anti-GFP antibody (Santa Cruz Biotechnology, Dallas, TX, USA). The peroxidase signal was visualized using Super Signal West Pico (Pierce Biotechnology, Waltham, MA, USA) on Super RX medical X-ray film (Fomei, Hradec Králové, Czech Republic). Membranes stained with Coomassie blue were used as loading controls (Figures S1 and S2).

### 4.6. RNA Isolation and Northern Blotting

Cell biomass of fractions harvested from giant colonies was suspended in TES buffer (10 mM Tris, pH 7.5, 10 mM EDTA, 0.5% SDS) and total RNA was isolated using the hot phenol method [15]. The total RNA concentration was quantified with a Qubit 2.0 Fluorometer (Invitrogen, Waltham, MA, USA). Fifteen milligrams of total RNA were denatured in loading buffer with formamide, separated in 1.5% agarose gel and then transferred to a positively charged nylon membrane (Amersham HybondTM-XL, GE Healthcare Ltd., Hatfield, UK). The membranes were hybridized with specific DNA probes prepared with a Random Primer DNA Labeling Kit Ver. 2 (Takara, Kusatsu, Japan) using the PCR fragments of particular genes and [α-32P] dCTP (MP Biomedicals, Santa Ana, CA, USA). The rRNA content was visualized by GelRed staining (Biotium, Fremont, CA, USA) and used as a loading control (Figure S3).

## Figures and Tables

**Table 1:** Strains.

Strain	Genotype	Source
BY4742	MATα, *his3*Δ1, *leu2*Δ0, *lys2*Δ0, *ura3*Δ0	Euroscarf
BY-*rtg1*	MATα, *his3*Δ1, *leu2*Δ0, *lys2*Δ0, *ura3*Δ0, *rtg1*::NatMX	[13]
BY-*mks1*	MATα, *his3*Δ1, *leu2*Δ0, *lys2*Δ0, *ura3*Δ0, *mks1*::NatMX	[13]
BY-*rtg1mks1*	MATα, *his3*Δ1, *leu2*Δ0, *lys2*Δ0, *ura3*Δ0, *rtg1*::hph, *mks1*::NatMX	[13]
BY-*rtg2mks1*	MATα, *his3*Δ1, *leu2*Δ0, *lys2*Δ0, *ura3*Δ0, *rtg2*::hph, *mks1*::NatMX	[13]
BY-Arg1p-GFP	MATα, *his3*Δ1, *leu2*Δ0, *lys2*Δ0, *ura3*Δ0, *ARG1*-yEGFP-KanMX	this study
BY-*rtg1*-Arg1p-GFP	MATα, *his3*Δ1, *leu2*Δ0, *lys2*Δ0, *ura3*Δ0, *rtg1*::NatMX, *ARG1*-yEGFP-KanMX	this study
BY-*mks1*-Arg1p-GFP	MATα, *his3*Δ1, *leu2*Δ0, *lys2*Δ0, *ura3*Δ0, *mks1*::NatMX, *ARG1*-yEGFP-KanMX	this study
BY-Cat2p-GFP	MATα, *his3*Δ1, *leu2*Δ0, *lys2*Δ0, *ura3*Δ0, *CAT2*-yEGFP-KanMX	this study
BY-*rtg1*-Cat2p-GFP	MATα, *his3*Δ1, *leu2*Δ0, *lys2*Δ0, *ura3*Δ0, *rtg1*::NatMX, *CAT2*-yEGFP-KanMX	this study
BY-*mks1*-Cat2p-GFP	MATα, *his3*Δ1, *leu2*Δ0, *lys2*Δ0, *ura3*Δ0, *mks1*::NatMX, *CAT2*-yEGFP-KanMX	this study
BY-Cit3p-GFP	MATα, *his3*Δ1, *leu2*Δ0, *lys2*Δ0, *ura3*Δ0, *CIT3*-yEGFP-KanMX	this study
BY-*rtg1*-Cit3p-GFP	MATα, *his3*Δ1, *leu2*Δ0, *lys2*Δ0, *ura3*Δ0, *rtg1*::NatMX, *CIT3*-yEGFP-KanMX	this study
BY-*mks1*-Cit3p-GFP	MATα, *his3*Δ1, *leu2*Δ0, *lys2*Δ0, *ura3*Δ0, *mks1*::NatMX, *CIT3*-yEGFP-KanMX	this study
BY-Crc1p-GFP	MATα, *his3*Δ1, *leu2*Δ0, *lys2*Δ0, *ura3*Δ0, *CRC1*-yEGFP-KanMX	this study
BY-*rtg1*-Crc1p-GFP	MATα, *his3*Δ1, *leu2*Δ0, *lys2*Δ0, *ura3*Δ0, *rtg1*::NatMX, *CRC1*-yEGFP-KanMX	this study
BY-*mks1*-Crc1p-GFP	MATα, *his3*Δ1, *leu2*Δ0, *lys2*Δ0, *ura3*Δ0, *mks1*::NatMX, *CRC1*-yEGFP-KanMX	this study
BY-Dld3p-GFP	MATα, *his3*Δ1, *leu2*Δ0, *lys2*Δ0, *ura3*Δ0, *DLD3*-yEGFP-KanMX	this study
BY-*rtg1*-Dld3p-GFP	MATα, *his3*Δ1, *leu2*Δ0, *lys2*Δ0, *ura3*Δ0, *rtg1*::NatMX, *DLD3*-yEGFP-KanMX	this study
BY-*mks1*-Dld3p-GFP	MATα, *his3*Δ1, *leu2*Δ0, *lys2*Δ0, *ura3*Δ0, *mks1*::NatMX, *DLD3*-yEGFP-KanMX	this study
BY-Gdh3p-GFP	MATα, *his3*Δ1, *leu2*Δ0, *lys2*Δ0, *ura3*Δ0, *GDH3*-yEGFP-KanMX	this study
BY-*rtg1*-Gdh3p-GFP	MATα, *his3*Δ1, *leu2*Δ0, *lys2*Δ0, *ura3*Δ0, *rtg1*::NatMX, *GDH3*-yEGFP-KanMX	this study
BY-*mks1*-Gdh3p-GFP	MATα, *his3*Δ1, *leu2*Δ0, *lys2*Δ0, *ura3*Δ0, *mks1*::NatMX, *GDH3*-yEGFP-KanMX	this study
BY-Leu1p-GFP	MATα, *his3*Δ1, *leu2*Δ0, *lys2*Δ0, *ura3*Δ0, *LEU1*-yEGFP-KanMX	this study
BY-*rtg1*-Leu1p-GFP	MATα, *his3*Δ1, *leu2*Δ0, *lys2*Δ0, *ura3*Δ0, *rtg1*::NatMX, *LEU1*-yEGFP-KanMX	this study
BY-*mks1*-Leu1p-GFP	MATα, *his3*Δ1, *leu2*Δ0, *lys2*Δ0, *ura3*Δ0, *mks1*::NatMX, *LEU1*-yEGFP-KanMX	this study
BY-Oac1p-GFP	MATα, *his3*Δ1, *leu2*Δ0, *lys2*Δ0, *ura3*Δ0, *OAC1*-yEGFP-KanMX	this study
BY-*rtg1*-Oac1p-GFP	MATα, *his3*Δ1, *leu2*Δ0, *lys2*Δ0, *ura3*Δ0, *rtg1*::NatMX, *OAC1*-yEGFP-KanMX	this study
BY-*mks1*-Oac1p-GFP	MATα, *his3*Δ1, *leu2*Δ0, *lys2*Δ0, *ura3*Δ0, *mks1*::NatMX, *OAC1*-yEGFP-KanMX	this study
BY-Pdh1p-GFP	MATα, *his3*Δ1, *leu2*Δ0, *lys2*Δ0, *ura3*Δ0, *PDH1*-yEGFP-KanMX	this study
BY-*rtg1*-Pdh1p-GFP	MATα, *his3*Δ1, *leu2*Δ0, *lys2*Δ0, *ura3*Δ0, *rtg1*::NatMX, *PDH1*-yEGFP-KanMX	this study
BY-*mks1*-Pdh1p-GFP	MATα, *his3*Δ1, *leu2*Δ0, *lys2*Δ0, *ura3*Δ0, *mks1*::NatMX, *PDH1*-yEGFP-KanMX	this study
BY-Tah1p-GFP	MATα, *his3*Δ1, *leu2*Δ0, *lys2*Δ0, *ura3*Δ0, *TAH1*-yEGFP-KanMX	this study
BY-*rtg1*-Tah1p-GFP	MATα, *his3*Δ1, *leu2*Δ0, *lys2*Δ0, *ura3*Δ0, *rtg1*::NatMX, *TAH1*-yEGFP-KanMX	this study
BY-*mks1*-Tah1p-GFP	MATα, *his3*Δ1, *leu2*Δ0, *lys2*Δ0, *ura3*Δ0, *mks1*::NatMX, *TAH1*-yEGFP-KanMX	this study
BY-Yat1p-GFP	MATα, *his3*Δ1, *leu2*Δ0, *lys2*Δ0, *ura3*Δ0, *YAT1*-yEGFP-KanMX	this study
BY-*rtg1*-Yat1p-GFP	MATα, *his3*Δ1, *leu2*Δ0, *lys2*Δ0, *ura3*Δ0, *rtg1*::NatMX, *YAT1*-yEGFP-KanMX	this study
BY-*mks1*-Yat1p-GFP	MATα, *his3*Δ1, *leu2*Δ0, *lys2*Δ0, *ura3*Δ0, *mks1*::NatMX, *YAT1*-yEGFP-KanMX	this study
BY-Yat2p-GFP	MATα, *his3*Δ1, *leu2*Δ0, *lys2*Δ0, *ura3*Δ0, *YAT2*-yEGFP-KanMX	this study
BY-*rtg1*-Yat2p-GFP	MATα, *his3*Δ1, *leu2*Δ0, *lys2*Δ0, *ura3*Δ0, *rtg1*::NatMX, *YAT2*-yEGFP-KanMX	this study
BY-*mks1*-Yat2p-GFP	MATα, *his3*Δ1, *leu2*Δ0, *lys2*Δ0, *ura3*Δ0, *mks1*::NatMX, *YAT2*-yEGFP-KanMX	this study

## Data Availability

Publicly available datasets were analyzed in this study. This data can be found here: http://www.ebi.ac.uk/pride/archive/projects/ PXD026077.

## Supplementary Materials

Supplementary materials can be found at https://www.mdpi.com/article/10.3390/ijms22115597/s1.
