# Supplementary material for: Mitochondrial Retrograde Signaling Contributes to Metabolic Differentiation in Yeast Colonies
Source: Int J Mol Sci. 2021 May 25;22(11):5597. doi: 10.3390/ijms22115597 (PMC8198273; doi:10.3390/ijms22115597)
Supplement: Supplementary file 1 [file ijms-22-05597-s001.zip › Table S4.pdf]

Table S4. List of the primers

**Primer Name**	**Primer sequence**
ARG1-GFP FW	AAATACGGTGAATCCAAAAAAACCAAAGGTGAAGAGTTGACTTTGGGTGACGGTGCTGGTTTA
ARG1-GFP RV	GGAGGAAGAGATCGTTATCTATCTTGAGGCGATGAACTAGCGGACTCGATGAATTCGAGCTCG
CAT2-GFP FW	ATTTTTGACGCCTTGGAAAATGAGAATAAACGAAAAGCAAAGTTAGGTGACGGTGCTGGTTTA
CAT2-GFP RV	CAAAAATATTCACAAATTAATTGAAGAGGAAAGGTGAAAAATTCATCGATGAATTCGAGCTCG
CIT3-GFP FW	TCTGGAAGCACTTACCAAAGCAAGCAATGTTAACAAGTTGGGTGACGGTGCTGGTTTA
CIT3-GFP RV	TGTAATAGCAAACGAAGCATTCTTGTAAATTGGAACTGCGTCGATGAATTCGAGCTCG
CRC1-GFP FW	TTAGGTGTGGAGATGACTCATTCACTGTTCAAGAAGTATGGCATAGGTGACGGTGCTGGTTTA
CRC1-GFP RV	CCAGAAAAAAATGGGAGGCAGGTCCACCTAACAAAAATTTAATCATCGATGAATTCGAGCTCG
DLD3-GFP FW	AATCACTACGATCCAAATGGAATCTTAAACCCATACAAGTACATTGGTGACGGTGCTGGTTTA
DLD3-GFP RV	TAAAGAAAAAGGGTTTGCTCTTTGAAAGTTAAAAATTAACAAAGTTCGATGAATTCGAGCTCG
GDH3-GFP FW	TTCGTCATGGTGGCTGACGCAATGCTTGACCAGGGAGACGTTTTTGGTGACGGTGCTGGTTTA
GDH3-GFP RV	CACTACTACATACACAGATAGTTACGAACAAAAAGAAAATAGCGCTTACGGTCGATGAATTCGAGCTCG
LEU1-GFP FW	AGAAAAGCTGTTACAACTACTTTCGATAAAGTCCACCAGGATTGGGGTGACGGTGCTGGTTTA
LEU1-GFP RV	AGACACATGTTATTGACGCCAGGTTTGGACGTTGTTTTTCACTGTTCGATGAATTCGAGCTCG
OAC1-GFP FW	ATGAAACTAGTTTATTCGATAGAGTCGAGAGTTTTAGGCCATAATGGTGACGGTGCTGGTTTA
OAC1-GFP RV	CTGGCCAATGAATGAAACTTCAAACCTCGGAGTTTGTTATGGGAATCGATGAATTCGAGCTCG
PDH1-GFP FW	TTGAATATGCAAATCGACAAATACATGGATTTGTTTACTGAGGGAGGTGACGGTGCTGGTTTA
PDH1-GFP RV	AATTGAAGTAGGTACCATGCCGTTCGCTCTTAAAGAGCCAACCATTCGATGAATTCGAGCTCG
TAH1-GFP FW	CCTGTTGTAGAGGTTGATGAACTACCGGAGGGATACGACCGGTCCGGTGACGGTGCTGGTTTA
TAH1-GFP RV	TATATAATTTTAAAGATAAATTAGAAGTGTTCCCTTTCTCTTTTCTCGATGAATTCGAGCTCG
YAT1-GFP FW	TACCTGTTGTCCGGCTACGATTACTTCGACGTGAGCGTGTCCGGTGGTGACGGTGCTGGTTTA
YAT1-GFP RV	AGTAGTAAAAATAATATTGATGCGCAAAAACTCAGCATAAACTCATCGATGAATTCGAGCTCG
YAT2-GFP FW	TTTGACCGCAGTCGTGTGGGTAGAAAGGTGGCGACCTTAGATCAAGGTGACGGTGCTGGTTTA
YAT2-GFP RV	GCTTACGCATAATGCTAGTAATAAATAGATAAACAAAGAGCGTTCTCGATGAATTCGAGCTCG
RTG1 DEL FW	CAAAAAACACTAGATAGTGAACCAAAAGAAAGCACAACACCAAACCAGCTGAAGCTTCGTACGC
RTG1 DEL RV	ACGAGGGTTATCACAACATAGCAATAGTGAGAGTCAGAAGTACTTGCATAGGCCACTAGTGGATCTG
MKS1 DEL FW	AACACTTCCTAATTATTCTCTAATCCTAATAAAAAAAAAGAACTGCAGCTGAAGCTTCGTACGC
MKS1 DEL RV	TTGAAAGAACTTTAAATACTGTATCTGATTTATTTAACTTAGTAAGCATAGGCCACTAGTGGATCTG
