# Supplementary material for: Mitochondrial Retrograde Signaling Contributes to Metabolic Differentiation in Yeast Colonies
Source: Int J Mol Sci. 2021 May 25;22(11):5597. doi: 10.3390/ijms22115597 (PMC8198273; doi:10.3390/ijms22115597)
Supplement: Supplementary file 1 [file ijms-22-05597-s001.zip › Figures S1-S2-S3-R2.pdf]

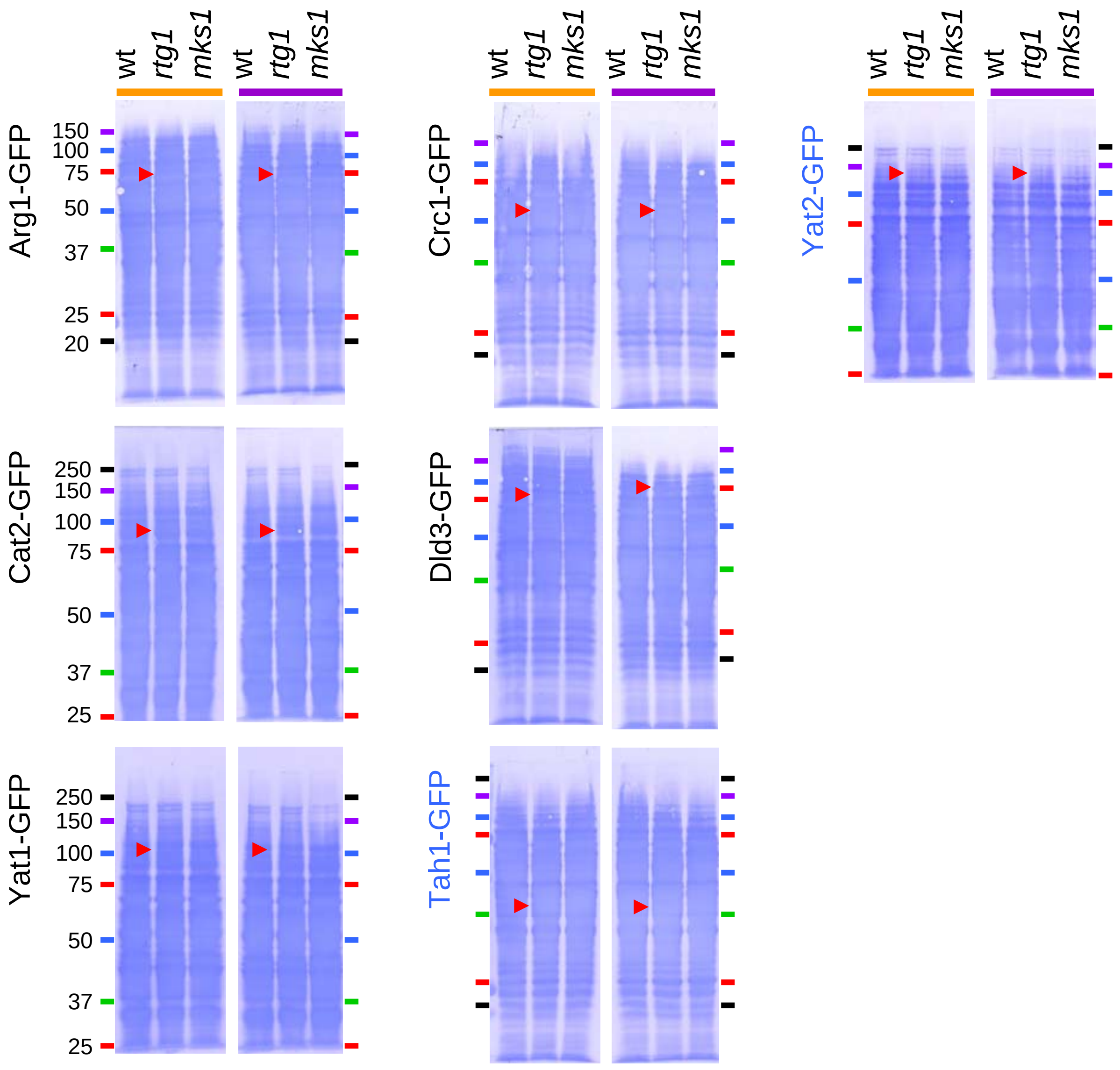

Figure S1: Western blot loading controls. Loading controls for Figure 3A. Arrows indicate the position of a particular protein on a Western blot.

Giant colonies

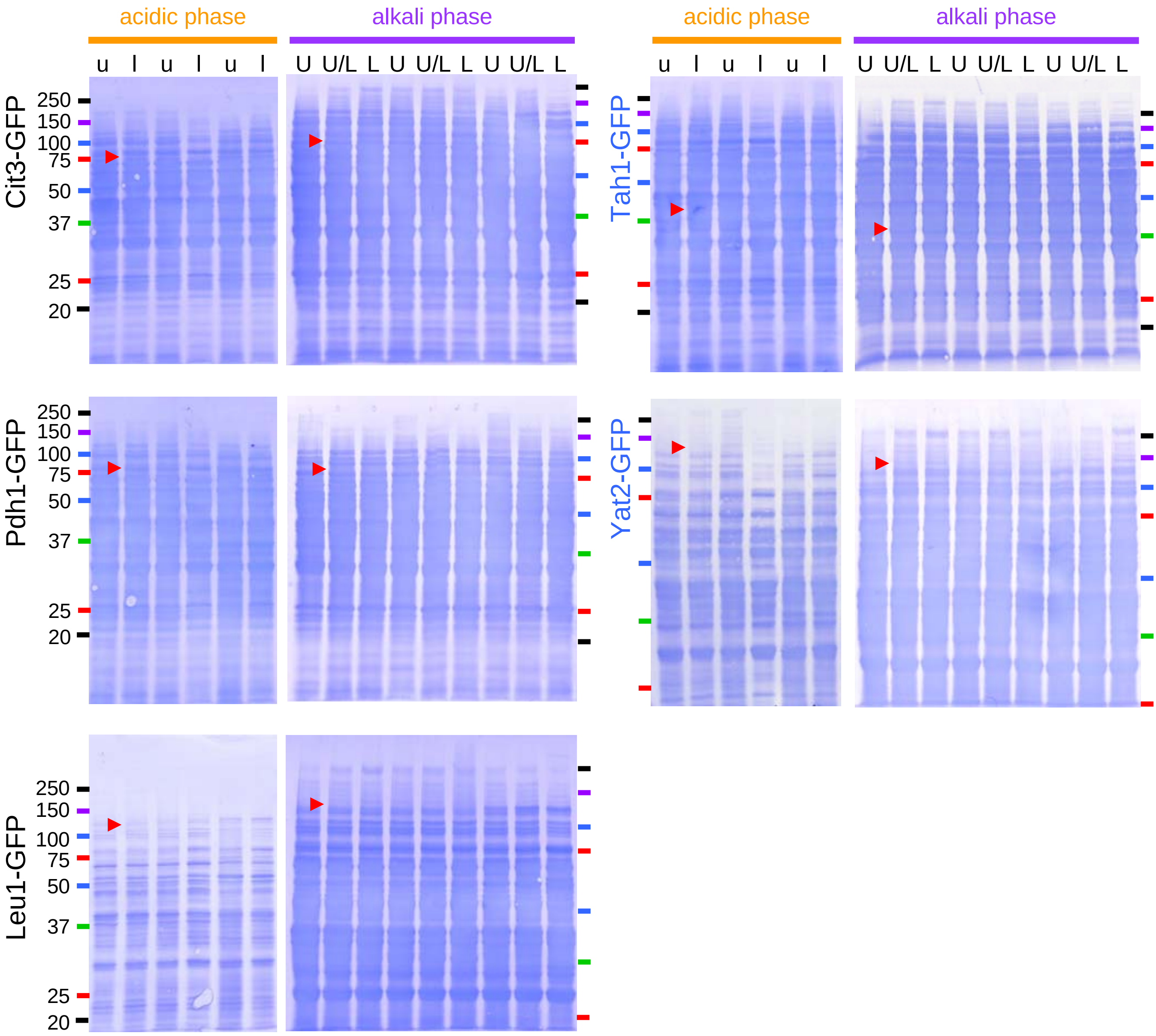

Figure S2: Western blot loading controls. Loading controls for Figure 3B. Arrows indicate the position of a particular protein on a Western blot.

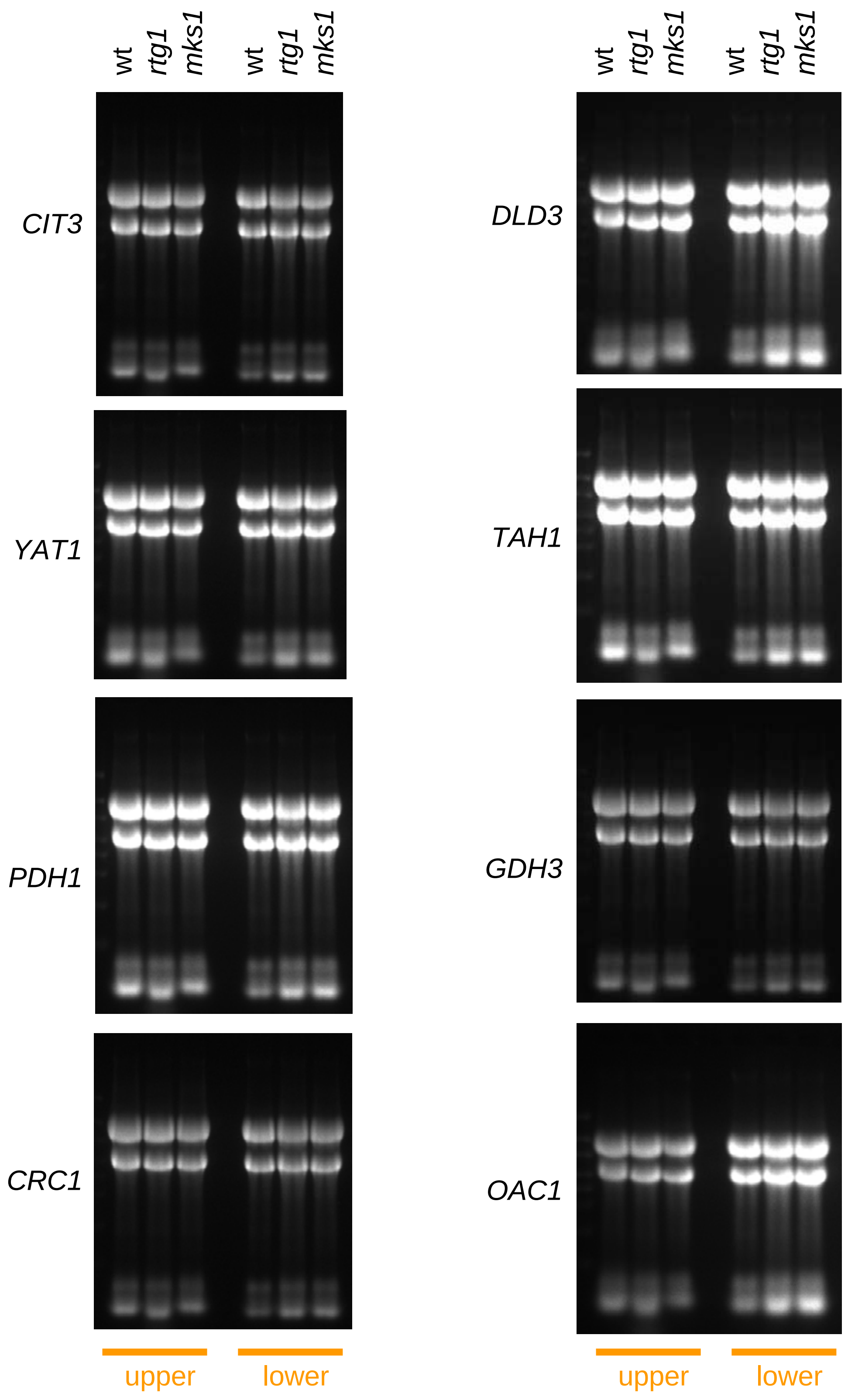

Figure S3: Northern blot loading controls. Loading controls for Figure 5.
